# Absence of Oral Opportunistic Infections in Patients with Inflammatory Bowel Disease Receiving Anti-TNF-α and Anti-Integrin-α_4_β_7_ Therapy

**DOI:** 10.3390/dj10030032

**Published:** 2022-02-23

**Authors:** Ema Saltović, Brankica Mijandrušić-Sinčić, Alen Braut, Ivana Škrobonja, Ella Sever, Irena Glažar, Sonja Pezelj-Ribarić, Miranda Muhvić-Urek

**Affiliations:** 1 Clinic of Dental Medicine, Clinical Hospital Center Rijeka, Krešimirova 40, 51000 Rijeka, Croatia; ema.saltovic@uniri.hr (E.S.); alen.braut@fdmri.uniri.hr (A.B.); irena.glazar@fdmri.uniri.hr (I.G.); sonja.pezelj.ribaric@fdmri.uniri.hr (S.P.-R.); 2 Clinic of Internal Medicine, Clinical Hospital Center Rijeka, Krešimirova 42, 51000 Rijeka, Croatia; brankica.sincic@medri.uniri.hr; 3 Department of Internal Medicine, Faculty of Medicine, University of Rijeka, Braće Branchetta 20/1, 51000 Rijeka, Croatia; 4 Department of Restorative Dentistry and Endodontics, Faculty of Dental Medicine, University of Rijeka, Krešimirova 40, 51000 Rijeka, Croatia; 5 Clinical Department for Clinical Microbiology, Clinical Hospital Center Rijeka, Krešimirova 42, 51000 Rijeka, Croatia; ivana.skrobonja@ri.t-com.hr; 6 Department of Oral Medicine and Periodontology, Faculty of Dental Medicine, University of Rijeka, Krešimirova 40, 51000 Rijeka, Croatia; ella.sever@uniri.hr; 7 Department of Dental Medicine, Faculty of Dental Medicine and Health, Josip Juraj Strossmayer University of Osijek, Crkvena 21, 31000 Osijek, Croatia

**Keywords:** infections, opportunistic, inflammatory bowel disease, oral candidiasis, therapy, biological

## Abstract

Biological therapy of inflammatory bowel disease (IBD) carries an increased risk for the development of opportunistic infections due to immunomodulation. The aim of this study was to determine the prevalence and types of oral infections in IBD patients treated with biological (anti-TNF-α and anti-integrin-α_4_β_7_) and conventional medication protocols. The study included 20 IBD patients receiving anti-TNF-α therapy, 20 IBD patients receiving anti-integrin-α_4_β_7_ therapy and 20 IBD patients without immunomodulatory therapy. Participants completed questionnaires on medical information, oral lesions and symptoms. For each patient, clinical examination and a salivary flow rate test were performed, followed by a swab of the oral mucosa. The swab samples were cultured to identify *Candida* spp. and oral bacteria. No bacterial opportunistic infections were detected. Candidiasis was detected in four participants, with no significant difference between groups (*p* = 0.765). Hyposalivation was most common in the anti-TNF-α group, with a significant difference between groups (*p* = 0.036). There were no significant differences between groups in self-reported oral mucosal lesions and symptoms (*p* > 0.05), or in the distribution of oral mucosal lesions (*p* > 0.05). This study suggests that IBD patients receiving biological therapy are at no greater risk of developing oral opportunistic infections than IBD patients not receiving immunomodulatory therapy.

## 1. Introduction

Over the past decade, the treatment of inflammatory bowel disease (IBD) has become increasingly dependent on immunomodulation, including biological therapy [1,2]. Given the immunomodulatory properties, the potential for the development of opportunistic infections has increased [1,2]. Opportunistic infections are defined as potentially progressive infections caused by a microorganism that usually has no pathogenic ability to cause infection in an immunocompetent host, but can cause a serious disease under the circumstances of a compromised immune system [1,3,4,5].

The goal of treating IBD with biological therapy is not only to control symptoms but also to modify the progression of the disease and preserve the intestinal function [6]. Anti-tumor necrosis factor alpha (anti-TNF-α) agents were the first to be described and used as biologics for the treatment of IBD, but because of the complex pathophysiology of the disease, their efficacy has not been demonstrated in all patients [6]. The development of new non-TNF-α biologics with a different mechanism of action has helped to overcome the negatives. This group includes anti-integrins, anti-interleukin (IL) 12/23 and anti-Janus kinase (JAK). The agents currently approved for the treatment of IBD are infliximab, adalimumab, golimumab and certolizumab pegol as anti-TNF-α agents, natalizumab and vedolizumab as anti-integrin agents, ustekinumab as an anti-IL 12/23 agent [7] and tofacitinib as an anti-JAK agent [8]. Unlike other biologic therapeutics that have a systemic mechanism of action, anti-integrin-α_4_β_7_ is considered to be gut-selective, meaning that its action is primarily limited to the site of inflammation in the gastrointestinal tract [9].

Although it has been suggested that the risk of opportunistic infections is increased with anti-TNF-α therapy, recent studies have shown that the risks have varied, while vedolizumab, for example, has not been associated with major infectious complications because of its primary gut selectivity [10].

In addition to immunomodulatory therapy, IBD patients are at increased risk for opportunistic infections because of age, comorbidity, reduced nutritional status, parenteral nutrition and bowel surgery [1], of which malnutrition is the major risk factor for infections development [11].

Despite the numerous research papers on infections in IBD patients receiving biological therapy [3,4,10], no study has addressed the oral infections in these patients.

The present study aimed to determine the prevalence and types of oral infections in patients with inflammatory bowel disease treated with biological therapy (anti-TNF-α and anti-integrin-α_4_β_7_) and conventional therapy. Our hypothesis is that patients receiving anti-TNF-α therapy have more infections than patients receiving anti-integrin-α_4_β_7_ or conventional therapy.

## 2. Materials and Methods

### 2.1. Participants

A group of IBD patients (*n* = 60) from the Department of Internal Medicine, Clinical Hospital Center Rijeka, were asked to participate in this study. Patients were diagnosed with Crohn’s disease (CD) or ulcerative colitis (UC) according to clinical, radiological, endoscopic and histopathological criteria described in the European Crohn’s and Colitis Organization (ECCO) guidelines [12,13]. The inclusion criteria were age of 18 and older, UC and CD patients treated with biological anti-TNF-α or anti-integrin-α_4_β_7_ therapy for more than three months and UC and CD patients treated with non-immunosuppressive therapy. The exclusion criteria were UC and CD patients treated with other forms of biological therapy and immunosuppressive therapy (>20 mg prednisone, methotrexate and azathioprine), intermittent smokers, smoking fewer than five cigarettes per day, systemic or topical antifungal therapy, antibiotics used in the previous month and/or oral mouth rinses used in the previous month.

Participants were divided into three groups: patients receiving anti-TNF-α biological therapy (*n* = 20), patients receiving anti-integrin-α_4_β_7_ biological therapy (*n* = 20) and patients receiving conventional treatment (*n* = 20) without any immunomodulatory therapy.

### 2.2. Questionnaire

Participants were interviewed using a questionnaire that included information on age, sex, type of IBD, other diseases, prescribed medications, smoking status (more than five cigarettes per day), denture wearing and the following oral complaints and symptoms (in the past year for the conventional group and in the period of biological therapy for the anti-TNF-α and anti-integrin-α_4_β_7_ groups): dry mouth, burning sensation, taste changes, halitosis, presence of lesions in the mouth and labial herpes.

### 2.3. Clinical Examination

Clinical examinations were performed in the Department of Oral Medicine of the Dental Clinic of the Clinical Hospital Center Rijeka. Clinical data were collected from the patient sitting in a dental chair illuminated with a professional dental light and using a set of standardized dental instruments. Intraoral examinations were performed by one of the authors (E.S.). Oral infections were diagnosed using the clinical features listed in the World Health Organization guidelines [14] and the Burket’s Oral Medicine textbook [15], as well as microbiological analyses. A salivary flow rate test for whole unstimulated saliva was performed by the spitting method for five minutes and hyposalivation was diagnosed when the salivary flow rate was under 1 mL per five minutes. Other oral lesions were recorded [14,15] and biopsied when necessary.

### 2.4. Cultivation and Identification of Candida spp. and Oral Bacteria

Samples were collected with sterile swabs and cultured on Sabouraud dextrose agar at 37 °C for 48 h. All isolates were identified by germ tube production, grown on a selective nutrient medium containing chromogenic enzyme substrates (HICromeTM Candida Differential Agar, HiMedia, Mumbai, India) and the Vitek 2 YST (bio-Merieux, Marcy-l’Etoile, France) automated identification system [16].

Conventional in vitro culture techniques were used for bacterial-specific identification. Swab material was cultured aerobically on agar containing 5% defibrinated sheep blood (Blood Agar Base, Biolab, Budapest, Hungary) at 37 °C for 48 h. Simple biochemical tests and the automated identification system Vitek 2 (bio-Merieux, Marcy-l’Etoile, France) were used to distinguish pathogenic bacteria from the microflora of the healthy oral cavity.

### 2.5. Laboratory Tests and Activity of the Disease

The medical data on patient history and medication were taken from the hospital’s information system. Laboratory tests were performed in all patients, including blood glucose and iron level, C-reactive protein (CRP) level and fecal calprotectin level. Clinical activity of the disease was assessed using the Harvey–Bradshaw index (HBI) for CD and the Mayo score for UC.

### 2.6. Statistical Analysis

Statistical analysis of the data was performed using JASP for Windows, version 0.14.1.0 (Amsterdam, The Netherlands). The Shapiro–Wilk test was used to test the normality of the distribution. The chi-square test was used to test the differences between the groups in terms of sex, type of disease, prevalence of candidiasis and hyposalivation, self-reported oral lesions and symptoms and distribution of oral mucosal lesions. ANOVA was used to determine the differences between the groups in terms of age and duration of biological therapy, and the Kruskal–Wallis test in terms of duration of disease, calprotectin and CRP values. A *p*-value < 0.05 was considered statistically significant.

### 2.7. Ethical Considerations

The study protocol was approved by the Ethical Committee of the Clinical Hospital Center Rijeka (Ethical approval code 003-05/20-1/41). The ethical guidelines of the Declaration of Helsinki were followed. All participants provided their informed consent before being enrolled in the study.

## 3. Results

### 3.1. Demographic and Disease Data

The demographic and disease data of the participants are shown in Table 1. No statistically significant differences were found between the groups in terms of sex, age, type of disease, CRP levels, fecal calprotectin levels and duration of biological therapy (*p* > 0.05). A statistically significant difference was found between the groups in terms of duration of disease (*p* = 0.005). Of the participants with CD, 11 had clinically active disease (HBI score > 5), while 20 were in clinical remission (HBI score < 5). Five participants with UC had clinically active disease (Mayo score > 2), while 24 were in clinical remission (Mayo score < 2).

### 3.2. Predisposing Factors for Candidiasis

**Figure 1:**
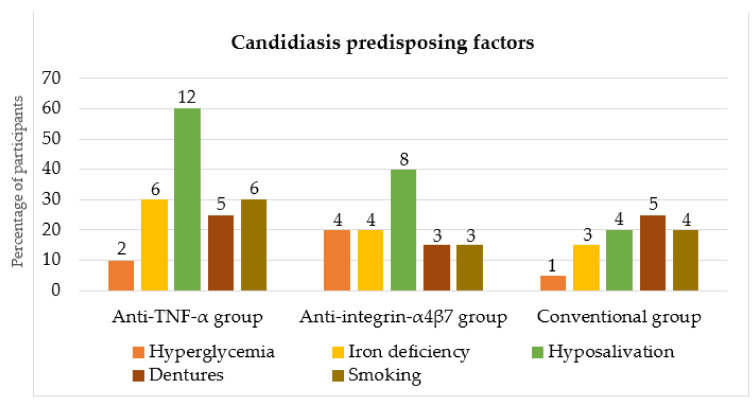
Predisposing factors for the development of candidiasis in the anti-TNF-α, anti-integrin-α_4_β_7_ and conventional groups (numbers above columns represent numbers of participants). TNF: tumor necrosis factor.

The evaluated predisposing factors for the development of candidiasis were elevated glucose levels and iron deficiency (systemic factors), and hyposalivation, denture wearing and smoking (local factors). The results of the predisposing factors of each group are shown in Figure 1. Hyposalivation was the most prevalent in the anti-TNF-α group, with a significant difference between the groups (*p* = 0.036).

### 3.3. Oral Opportunistic Infections

No bacterial opportunistic infections were found in the observed participants. Candidiasis was found in four participants (6.66%), predominantly *C. albicans* infection followed by *C. glabrata*, *C. krusei* and *C. dubliniensis,* as shown in Table 2. One patient in the anti-TNF-α group exhibited infection by *C. albicans*, with others showing infection by *C. albicans*, *C. krusei* and *C. dubliniensis*. Clinically, candidiasis presented as denture-associated erythematous stomatitis in three participants and as angular cheilitis in one participant. According to the Newton classification scale, candidiasis was observed as type I in two participants and type II in one participant. The duration of biological therapy in participants with candidiasis in the anti-TNF-α group was five years in one participant and six months in the other participant, whereas the duration of treatment in the anti-integrin-α_4_β_7_ group was four years. All participants with oral candidiasis were in clinical remission. The ages of participants with oral candidiasis were 64 in the conventional group, 70 and 82 in the anti-TNF-α group and 35 in the anti-integrin-α_4_β_7_ group. Predisposing factors for candidiasis in the four participants with candidiasis were hyposalivation (*n* = 4), dentures (*n* = 3), smoking (*n* = 1), elevated glucose level (*n* = 1) and iron deficiency (*n* = 1) (data shown in Appendix A, Table A1).

### 3.4. Self-Reported Oral Lesions and Symptoms

**Figure 2:**
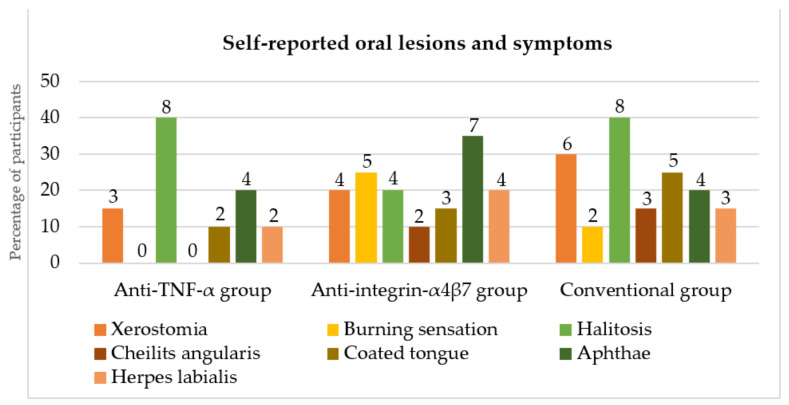
Self-reported oral lesions and symptoms in the anti-TNF-α, anti-integrin-α_4_β_7_ and conventional groups (numbers above columns represent numbers of participants). TNF: tumor necrosis factor.

The self-reported oral lesions and symptoms are shown in Figure 2. Participants in the anti-TNF-α and conventional groups most frequently reported halitosis, while aphthae were mostly reported in the anti-integrin-α_4_β_7_ group. There were no significant differences in self-reported oral lesions and symptoms between the groups (*p* > 0.05). None of the participants reported changes in taste sensation.

### 3.5. Oral Mucosal Lesions

**Figure 3:**
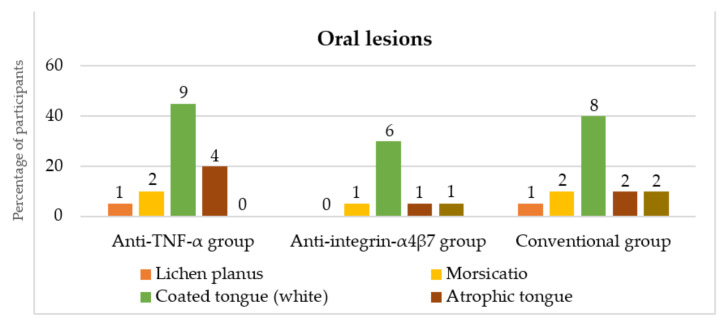
Distribution of oral mucosal lesions presented during the clinical examination in the anti-TNF-α, anti-integrin-α_4_β_7_ and conventional groups (numbers above columns represent numbers of participants). TNF: tumor necrosis factor.

The distribution of the oral mucosal lesions detected during the clinical examination is summarized in Figure 3. The most common lesion in all groups was coated tongue. No specific IBD lesions were found. There were no significant differences in the distribution of oral mucosal lesions between the groups (*p* > 0.05).

## 4. Discussion

Treatment of IBD with biologics has led to a significant reduction in symptoms and progression of inflammatory processes, resulting in a marked improvement in quality of life. Despite the benefits, biological therapies have increased the risk of opportunistic infections. This is not only dependent on the immunomodulatory therapy but can also be increased by known risk factors [17]. A large meta-analysis examining patients on biological therapy for rheumatoid arthritis concluded that biologic agents are associated with a slightly increased but significant risk of opportunistic infections [18].

To our knowledge, our study is the first to investigate the occurrence of oral opportunistic infections in IBD patients receiving anti-TNF-α and anti-integrin-α_4_β_7_ biological therapy. The study was conducted only with participants who received anti-TNF-α and anti-integrin-α_4_β_7_, as these types of biologics are used more frequently than others. Participants receiving conventional therapy were included as a control group to rule out other known infection predisposing factors as well as disease- and treatment-associated risk factors in IBD patients.

Immunomodulatory therapy in IBD poses a safety concern given the elevated risk of opportunistic infections [18]. An association between the immunomodulatory therapy used and bacterial, viral, fungal and parasitic infections has been noted [1]. Toruner et al. [19] found that infliximab is much more commonly associated with fungal and mycobacterial infections, while corticosteroids are associated with fungal infections, particularly by *Candida* spp., and azathioprine is associated with viral infections. These medications are often prescribed concomitantly with a higher possibility of infection development because of cumulative immunosuppression [1,11,20].

Higher disease severity has been identified as a risk factor for the development of opportunistic infections [19], independent of the effects of combined immunosuppressive medications [21]. In our study, disease activity did not prove to be a risk factor.

It has been suggested that the duration of therapy is related to the risk of developing opportunistic infections, with the first year of anti-TNF-α therapy (and particularly the first few months) being associated with a higher risk [22,23]. As reported in our study, only one participant with oral candidiasis was treated with biologics for six months, whereas others were treated for several years. It is possible that for the participant who was in the first year of therapy, the duration of therapy was a risk factor.

As age is a risk factor for infection, 17% of patients over 60 years of age receiving vedolizumab have been found to develop an infection in the first year of treatment and 20% of patients receiving anti-TNF-α for IBD [24]. In our study, three out of four participants with oral candidiasis were over sixty years old and therefore age must be considered as a risk factor for the development of infection. Anti-TNF-α therapy is associated with an increased risk of infection; therefore, it is possible that vedolizumab will be used in the future as a first-line therapy in patients who are at higher risk, such as older patients [9].

Although immunomodulators have been reported to carry an increased risk of infection, the extent of this risk is not clear [1]. The odds of developing any infection in recipients of biological therapy have been identified as 19% in the meta-analysis by Bonovas et al. [25]. That same study also reported the incidence of opportunistic infections to be 1.10% in treatment groups and 0.58% in placebo groups [25].

There are conflicting data on whether some biologics, such as anti-TNF-α agents, carry a higher risk of developing opportunistic infections. To our knowledge, there have been no studies investigating oral opportunistic infections in IBD patients receiving biological therapy; however, several studies have investigated opportunistic infections in IBD patients with no reference to oral infections. In the meta-analysis by Ford et al. [26], anti-TNF-α agents were reported to double the risk of opportunistic infections. In the case-control study by Salmon et al. [27], treatment with infliximab and adalimumab was a risk factor for opportunistic infections. However, Fidder et al. [28] found no significant difference in infection rates between IBD patients treated with infliximab and IBD patients receiving conventional therapy, as reported in our study. In the meta-analysis conducted by Shah et al. [29], cases of opportunistic infections were numerically higher in the anti-TNF-α and anti-integrin groups than in the placebo groups, but the relative risk was not significantly increased between groups. A similar finding was reported by Bonovas et al. [25], that biologics increase the risk of opportunistic infections, with no difference between anti-TNF-α and anti-integrin agents. Although we did not find an increased risk of opportunistic infections between groups, our results also show no difference between these agents. Hindryckx et al. [3] have suggested that there is a slightly increased risk of opportunistic infections in patients treated with anti-TNF-α agents compared with patients treated with vedolizumab or ustekinumab, which has not been demonstrated in clinical trials in CD patients. No increased risk of opportunistic infections was observed in vedolizumab and ustekinumab treatments. Colombel et al. [30] also found no increased risk of infection from treatment with vedolizumab compared to the placebo group, which is consistent with the gut-selectivity mechanism of action of vedolizumab. This was supported in the meta-analysis by Luthra et al. [31], which found that the risk of opportunistic infections was not significantly increased among the gut-selective anti-integrins.

In the gastrointestinal tract, *Candida* spp. are considered part of the normal flora, but under certain conditions, such as immunosuppression, they can cause disease [32]. The spectrum of diseases ranges from local mucosal infections to systemic dissemination [32]. Ahmad and Khan [33] reported four *Candida* spp. associated with invasive infections, including *C. albicans*, *C. glabrata*, *C. parapsilosis* and *C. tropicalis*. Our finding of *C. albicans* and *C. glabrata* in swab samples concurs with the reported findings, while *C. parapsilosis* and *C. tropicalis* were not found in our samples. Corazza et al. [34] found that oral candidiasis in patients with psoriasis on anti-TNF-α therapy was caused by *C. albicans*, *C. glabrata* and *C. parapsilosis*, whereas in patients with psoriasis without immunosuppressive therapy, candidiasis was caused by *C. albicans*, *C. dubliniensis* and *C. krusei*. In our study, only the finding of *C. albicans* aligns with Corazza et al. [34]. We found an opposite distribution of the other *Candida* spp., except for *C. parapsilosis*, which we did not find at all. In the meta-analysis by Ford et al. [26], only six cases of oral or esophageal candidiasis were found in over 4000 patients, which the authors explained as possible under-reporting.

The limitations of our study are the relatively small sample size, the fact that not all types of biologics for the treatment of IBD were included and the absence of a control group in the form of IBD patients without therapy. A larger sample size and inclusion of all types of biological therapy should be considered in future studies.

## 5. Conclusions

In conclusion, our study suggests that IBD patients receiving biological therapy are at no greater risk of developing oral opportunistic infections than IBD patients not receiving immunomodulatory therapy. Anti-TNF-α therapy was also not shown to be associated with a higher risk of developing oral opportunistic infections than anti-integrin-α_4_β_7_ therapy. Future studies are needed to investigate oral opportunistic infections in patients receiving other types of biologics.

## Figures and Tables

**Table 1:** Demographic and disease data of the participants in the anti-TNF-α, anti-integrin-α4β7 and conventional groups.

	Anti-TNF-α	Groups	Conventional	*p*-Value
		Anti-Integrin-α_4_β_7_		
**Sex**				
Female/*n* (%)	9 (45)	9 (45)	10 (50)	0.935 ^a^
Male/*n* (%)	11 (55)	11 (55)	10 (50)	
**Age (years)**				
Average	47	46.25	46	0.980 ^b^
Standard deviation	18.01	15.28	15.68	
Minimum	21	20	19	
Maximum	82	68	76	
**Type of disease**				
Crohn’s disease/*n* (%)	13 (65)	11 (55)	7 (35)	0.154 ^a^
Ulcerative colitis/*n* (%)	7 (35)	9 (45)	13 (65)	
**Disease duration (years)**				
Median	7.5	11.5	7	0.005 ^c^
Range	33	36	14	
Minimum	2	4	1	
Maximum	35	40	15	
**C-reactive protein levels (mg/L)**				
Median	4.3	3.7	7.68	0.998 ^c^
Range	97.8	110.7	39.4	
Minimum	0.5	0.6	0.6	
Maximum	98.3	111.3	40	
**Fecal calprotectin levels (μg/g)**				
Median	319.5	136	275.3	0.975 ^c^
Range	1907	2717.09	872	
Minimum	20	21.91	20	
Maximum	1927	2739	892	
**Type of therapy/*n* (%)**				
	infliximab	vedolizumab 20 (100)	mesalazine	
	17 (85)		18 (90)	
	adalimumab		sulfasalazine	
	3 (15)		1 (5)	
			prednisone < 20 mg	
			1 (5)	
**Duration of biological therapy (years)**				
Average	2.86	2.23		0.297 ^b^
Standard deviation	2.36	1.31		
Minimum	0.5	0.5		
Maximum	7	4		

TNF: Tumor necrosis factor. ^a^ Chi-square test. ^b^ ANOVA. ^c^ Kruskal–Wallis test.

**Table 2:** Prevalence of candidiasis and types of *Candida* spp. in the anti-TNF-α, anti-integrin-α_4_β_7_ and conventional groups.

	Anti-TNF-α Group	Anti-Integrin-α_4_β_7_ Group	Conventional Group	*p*-Value
**Candidiasis/*n* (%)**	2 (10)	1 (5)	1 (5)	0.765 ^a^
**Types of**				
***Candida* spp. **				
*C. albicans*	+	+	+	
*C. glabrata*	−	−	+	
*C. krusei*	+	−	−	
*C. dubliniensis*	+	−	−	

TNF: Tumor necrosis factor. ^a^ Chi-square test.

## Data Availability

The data presented in this study are available from the corresponding author upon request. The data are not publicly available due to privacy.

## Appendix A

**Table A1:** Predisposing factors, demographic and therapy data in participants with candidiasis.

	Patient 1	Patient 2	Patient 3	Patient 4
**Group**	Anti-TNF-α	Anti-TNF-α	Anti-integrin-α_4_β_7_	Conventional
Sex	female	male	male	male
Age/years	82	70	35	64
Duration of biological therapy/years	0.5	5	4	
Hyperglycemia	+	−	−	−
Iron deficiency	−	−	+	−
Hyposalivation	+	+	+	+
Dentures	+	+	−	+
Smoking	−	−	+	−

TNF: Tumor necrosis factor.
